# *In-situ* catalyzation approach for enhancing the hydrogenation/dehydrogenation kinetics of MgH_2_ powders with Ni particles

**DOI:** 10.1038/srep37335

**Published:** 2016-11-16

**Authors:** M. Sherif El-Eskandarany, Ehab Shaban, Naser Ali, Fahad Aldakheel, Abdullah Alkandary

**Affiliations:** 1 Nanotechnology and Advanced Materials Program, Energy and Building Research Center, Kuwait Institute for Scientific Research Safat, 13109, Kuwait - State of Kuwait.

## Abstract

One practical solution for utilizing hydrogen in vehicles with proton-exchange fuel cells membranes is storing hydrogen in metal hydrides nanocrystalline powders. According to its high hydrogen capacity and low cost of production, magnesium hydride (MgH_2_) is a desired hydrogen storage system. Its slow hydrogenation/dehydrogenation kinetics and high thermal stability are the major barriers restricting its usage in real applications. Amongst the several methods used for enhancing the kinetics behaviors of MgH_2_ powders, mechanically milling the powders with one or more catalyst species has shown obvious advantages. Here we are proposing a new approach for gradual doping MgH_2_ powders with Ni particles upon ball milling the powders with Ni-balls milling media. This proposed is-situ method showed mutually beneficial for overcoming the agglomeration of catalysts and the formation of undesired Mg_2_NiH_4_ phase. Moreover, the decomposition temperature and the corresponding activation energy showed low values of 218 °C and 75 kJ/mol, respectively. The hydrogenation/dehydrogenation kinetics examined at 275 °C of the powders milled for 25 h took place within 2.5 min and 8 min, respectively. These powders containing 5.5 wt.% Ni performed 100-continuous cycle-life time of hydrogen charging/discharging at 275 °C within 56 h without failure or degradation.

Owing to the dramatic global environmental changes associated with man-made carbon dioxide emissions and the huge consumption of the limited resources of fossil fuels, developing alternate energy sources is important for a sustainable future. The increase in threats from global warming due to the consumption of fossil fuels requires our planet to adopt new strategies to harness the inexhaustible sources of energy^1^. Hydrogen is an energy carrier, which holds tremendous promise as a new clean energy option^2^,^3^. It is a convenient, safe, versatile fuel source that can be easily converted to a desired form of energy without releasing harmful emissions^4^,^5^. A key advantage of hydrogen is that when burned, carbon dioxide (CO_2_) is not produced.

Mg and Mg-based materials have opened promising concept for storing hydrogen in a solid-state matter^6^. The natural abundance, cheap price, operational cost effectiveness, light weight, and high hydrogen storage capacity (7.60 wt.%, 0.11 kg H_2_L^−^) are some advantages of Mg and Mg-based alloys making them desirable storage materials for research and development^7^. Since 1991, nanocrystalline MgH_2_ powders has been successfully produced near room temperature by reactive ball milling technique (RBM)^8^,^9^, using high-energy ball mill operated at hydrogen atmospheric pressure. Some major drawbacks found in MgH_2_ system that should be solved. Firstly, MgH_2_ shows a high thermal stability making the hydrogen releasing at moderate temperatures (below 300 °C) very difficult^2^,^10^. Secondly, MgH_2_ exhibits very slow kinetics of hydrogenation/dehydrogenation at temperatures less than 350 °C. Innumerable efforts have been tackled to improve the kinetics behavior of MgH_2_ by catalyzing the metal hydride powders with wide spectrum of mono, binary and multicatalytic systems. One of the earliest work proposed for improve MgH_2_ powders was achieved by Prof. R. Schulz and his team work in 1999^11^. In their work, MgH_2_ powders were catalyzed by ball milling with one of 3-d transition metal powders of Ti, V, Mn, Fe and Ni. Based on their results, Ti and V showed better catalytic effect for hydrogen absorption and desorption when compared with Ni. Furthermore, Hanada *et al*.^12^ reported very interesting results on catalyzing of MgH_2_ powders by small amount (1 mol. %) of Fe, Co, Ni and Cu nanoparticles. The as-mechanically doped MgH_2_/Ni powders obtained after a very short milling time (2 h) showed excellent hydrogenation/dehydrogenation kinetics properties and enjoyed high storage capacity (~6.5 wt.%)^12^. Since then, different schools have reported attractive results upon using pure elemental powders such as Al, Ti, Fe, Ni, Cu and Nb^13^, intermetallic compounds^14^,^15^, metastable big-cube Zr_2_Ni^16^, and metal/metal oxide binary nanocomposite^17^ for improving the kinetics of hydrogen absorption/desorption of MgH_2_.

Besides the metal, semimetal, and metallic metastable phases, hard metal oxide of Nb_2_O_5_^16^ and refractory material powders and, such as SiC^18^, and TiC^19^ find a space of applications as excellent kinetics modifier used successfully for improving the hydrogen absorption/desorption behaviors of MgH_2_ system. More recently, we demonstrated the first report of employing a metallic glassy Zr_70_Ni_20_Pd_10_ powders for enhancing the hydrogenation/dehydrogenation properties of MgH_2_ powders^20^. In general, it is agreed that mechanically-induced doping of MgH_2_ with the abrasive powders of hard phases such as carbides, oxides, intermetallic and metallic glassy alloys materials lead to fast grain refining of the MgH_2_ upon releasing the crystalline stored energy, leading to refine the MgH_2_ grains along their grain boundaries where superfine grains are formed. Such desirable fine grains with their short-distance grain boundaries always facilitate short diffusion path^20^, leading to fast diffusion of the hydrogen atoms^21^,^22^,^23^. Recently, Crivello *et al*. have introduced a useful review article discussing the several ways used for improving MgH_2_-based materials^24^.

Apart from the usual approach of doping the MgH_2_ powders by desired catalyst(s) particles then high-energy ball milling the mixture for certain time, here we show, an interesting approach for gradual doping the MgH_2_ powders with Ni particles upon using Ni-balls as milling media. Our process shows a superior effect of Ni particles that were worn out from the balls and gradually introduced into the MgH_2_ matrix. In addition, our proposed *in-situ* method showed mutually beneficial for overcoming the agglomeration of Ni particles that usually leads to a heterogeneous catalytic distribution into MgH_2_ matrix. Accordingly, our synthesized nanocomposite MgH_2_/5.5 wt.% Ni composite powders revealed fast hydrogenation/dehydrogenation processes, taking place at moderate temperature and low value of activation energy (75 kJ/mol).

## Results

### Structure

**Figure 1:**
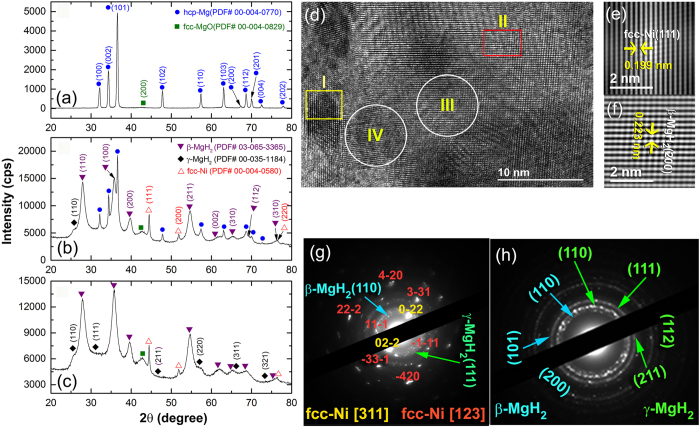
Structure change with changing the RBM time. XRD patterns of elemental hcp-Mg powders after RBM time for (**a**) 0 h, (**b**) 12.5 h, and (**c**) 25 h, using, using Ni-balls milling media. The FE-HRTEM micrograph of MgH_2_ powders obtained after 25 h of RBM, is shown in (**d**) together with the filtered lattice- resolution TEM images corresponding to (**e**) zone I, and (**f**) zone II. The NBDP and SADP related to zone III and zone IV are elucidated in (**g**) and (**h**), respectively.

X-ray diffraction (XRD) and field emission-high resolution transmission electron microscope (FE-HRTEM) techniques were employed to investigate the structural changes of hcp-Mg powders upon RBM under a hydrogen gas pressure (50 bar), using Ni-balls as milling media. The XRD pattern of elemental Mg powders (precursor) is shown in Fig. 1(a). The powders consisted of large polycrystalline grains, suggested by the sharp Bragg-peaks related to hcp-Mg (PDF file# 00-004-0770). After 12.5 h of RBM time, new Bragg-peaks corresponding to β-MgH_2_ (PDF file# 03-065-3365) and γ-MgH_2_ (PDF file# 00-035-1184) were appeared, implying the progress of a gas-solid reaction taking place between hydrogen gas and Mg powders (Fig. 1(b)). Besides these peaks, a new set of Bragg-lines became noticeable in the XRD presented in Fig. 1(b). Careful analysis of these lines indicated that they corresponding to fcc-Ni metal (PDF file# 00-004-0580) introduced to the powders due to employing of Ni-balls as milling media. Toward the end of RBM time (25 h); all the diffracted lines related to hcp-Mg were completely disappeared, suggesting the completion of RBM process (Fig. 1(c)). The broadening manifested in the Bragg peaks shown in Fig. 1(c) raised from both refinement of the MgH_2_ crystallites and accumulated macrostrain during the RBM process. Moreover, the Bragg-peaks of fcc-Ni metal maintained their peak positions after 25 h of RBM, implying the absence of Ni solubility into the MgH_2_ lattice, as elucidated in Fig. 1(c). These results comes in contrast to those ones demonstrated the formation of Mg_2_NiH_4_ phase during ball milling of MgH_2_ with Ni metal used as catalysts to improve the hydrogenation behavior of MgH_2_ (see for example ref. 11).

**Figure 2:**
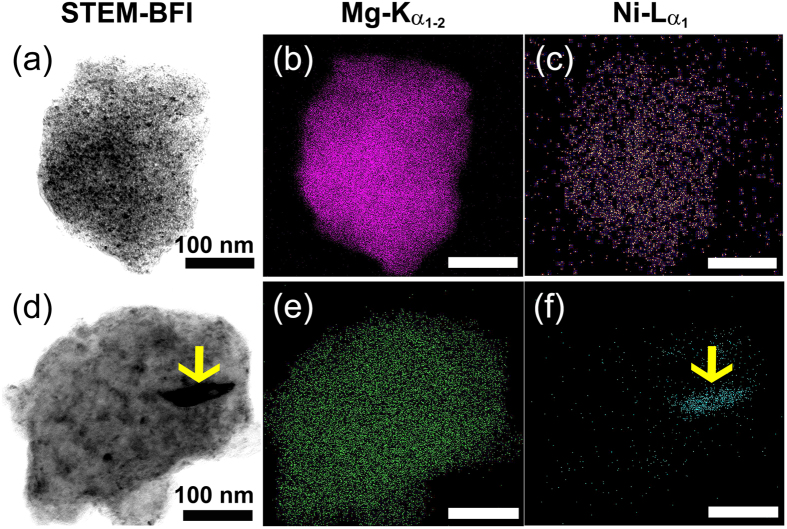
Morphological details and elemental distributions beyond the nanoscale level of MgH_2_ powders obtained after 25 h of RBM time. (**a**) STEM-BFI, (**b**) Mg-K_α1−2_ and (**c**) Ni-L_α1_ of the powders obtained after 25 h of RBM, using Ni-balls milling media. For comparison, the STEM image and corresponding EDS-elemental mapping of Mg and Ni for a different sample obtained after 25 h of RBM a mixture of MgH_2_ doped with 5.5 wt.% Ni nanoparticles are presented in (**d**), (**e**) and (**f**), respectively. The arrows shown in (**d**) and (**f**) refer to agglomerated Ni particles adhered onto the MgH_2_ aggregated powders.

The HRTEM micrograph of the powders obtained after 25 h of RBM is shown in Fig. 1(d). The powders revealed Moiré-like fringes with nanocrystalline-structure contained crystallites ranged in sizes between 5 nm to 17 nm in diameter (Fig. 2(a)). The filtered lattice resolution TEM image corresponding to zone I indexed in Fig. 1(d) is shown in Fig. 1(e). Obviously, the lattice fringes are regularly separated with an interplanar spacing (d) of 0.199 nm, which agrees well with the (111) lattice index of fcc-Ni metal (PDF file# 00-004-0770), as presented in Fig. 1(e). The filtered atomic resolution TEM image corresponding to zone II (Fig. 1(d)) is shown in Fig. 1(f). The clear Moiré-like fringes with d spacing of 0.223 nm (Fig. 1(f)) matches well with β-MgH_2_ (200) crystal. The nano beam diffraction pattern (NBDP) related to zone III implied precipitation of Ni crystals (oriented to zone axis [311] and [123]) coexisted with γ-MgH_2_ (111) and β-MgH_2_ (110) crystals, as elucidated in Fig. 1(g). Moreover, the corresponding selected area diffraction pattern (SADP) of zone IV (Fig. 1(h)) revealed continuous diffracted Debye-rings corresponding to β-MgH_2_ (110) and γ-MgH_2_ (111) phases. Based on careful TEM analysis performed for at least 50 tested zones of three individual samples, we could not detect the existence of any other phase(s) such as unprocessed Mg, Mg_2_Ni alloy and/or Mg_2_NiH_4_.

In a different set of experiment conducted under the same experimental conditions, MgH_2_ powders were obtained upon using FeCr-balls milling media. The XRD patterns of powder products of this set obtained after different RBM time are shown in the Supplementary Materials-Fig. S1. Since the hardness of FeCr alloy is higher than pure Ni metal, the FeCr particles coming from the balls were hardly seen in the sample obtained after 25 h, as displayed in Fig. S1(b). However, low intensity Bragg peaks related to FeCr alloy were detected after 50 h of RBM time, as presented in Fig. S1(c).

### Morphology

The morphological characterizations of the MgH_2_ powders obtained after 25 h of RBM time upon milling with Ni-balls were investigated by HRTEM/EDS-elemental mapping Fig. 2(a) shows the FE-scanning transmission electron microscope (STEM)/bright field (BF) image of MgH_2_ powders obtained after RBM of 25 h, using Ni-balls milling media. The STEM-BF image shows a typical composite aggregate (~300 nm in diameter) containing dark grey nano-spherical lenses (~5 nm in diameter) embedded into a light-gray matrix, as elucidated in Fig. 2(a). The corresponding STEM-EDS elemental mapping of MgK_α1−2_ confirmed that the light grey matrix was related to MgH_2_ phase (Fig. 2(b)), whereas those dispersoids nano-lenses shown in Fig. 2(a) were corresponding to Ni metal, as indicated by the STEM-EDS elemental mapping of Ni_α1−2_ (Fig. 2(c)). It should be notified that the spherical-like morphology nanocrystalline Ni with their spherical lens-like structure were homogeneously distributed into the over whole matrix of MgH_2_ beyond the nano-scale level, as displayed in Fig. 2(b) and (c).

In contrast, when the MgH_2_ powders were doped with 5.5 wt.% of Ni nanoparticles (~10 nm in diameter) and then ball milled for 25 h of RBM time using Cr-steel balls, the catalytic metal agent of Ni nanoparticles were agglomerated to form larger flaky-like particles of 90 nm in diameter, as indexed in Fig. 2(d). Based on the large size of these agglomerated Ni particles, they were heterogeneously distributed into the MgH_2_ matrix (Fig. 2(e)), tended to be located in one zone (Fig. 2(f)) into the matrix, where the other matrix zones were catalyst-free, as elucidated in Fig. 2(f). Growing of Ni metallic powders was attributed to the cold welding effect resulted from increasing the local temperature inside the vial, which was related to the action of the milling media during the milling process. However, when Ni balls were used, the Ni particles worn out from the balls were gradually introduced to the MgH_2_ matrix so that Ni catalysts had good opportunity to be distributed in a homogeneous fashion into the metal hydride matrix without serious agglomeration behavior (Fig. 2(c)).

### Thermal stability

**Figure 3:**
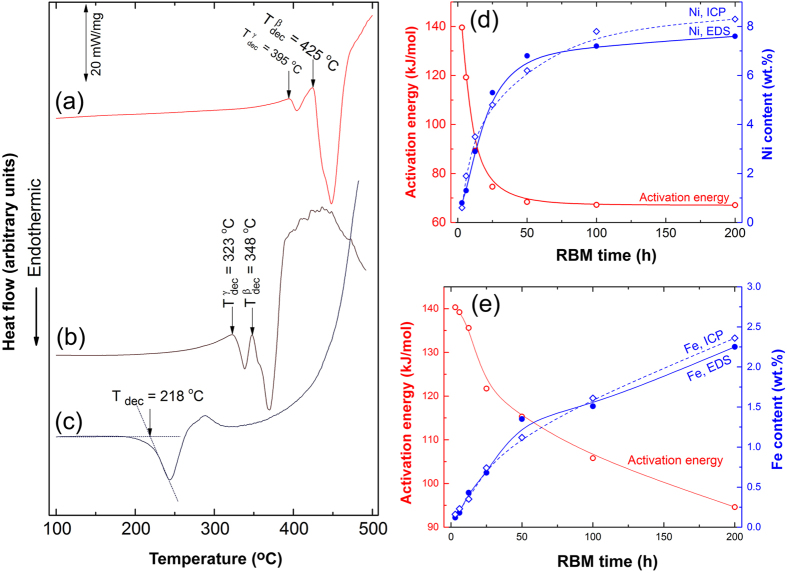
Thermal stability of MgH_2_ powders obtained after selected RBM time. DSC curves of MgH_2_ powders obtained after (**a**) 6 h, (**b**) 12.5 h, and (**c**) 25 h of RBM time, using Ni-balls milling media. Influence of RBM time on the activation energy and Ni concentration of MgH_2_ powders is displayed in (**d**). For comparison, the activation energy of the powders obtained upon using FeCr-balls milling media is plotted against FeCr concentration in (**e**) after different RBM time.

Differential scanning calorimetry (DSC) performed at a constant heating rate of 20 °C/min under a helium gas flow of 75 ml/min was employed to investigate the effect of RBM time and Ni concentration on the decomposition temperature (dehydrogenation temperature at normal pressure) of MgH_2_ powders. The DSC trace of as-synthesized MgH_2_ powders obtained after 6 h of RBM revealed two separated endothermic events at an onset temperature of 395 °C and 425 °C, as shown in Fig. 3(a). The XRD analysis of the powders that were individually heated up to 400 °C indicated the absence of γ-MgH_2_ phase, where β-MgH_2_ phase was remained. Therefore, it can be concluded that the first endothermic reaction peak referred to the decomposition process of γ-MgH_2_ metastable phase. In contrast, the XRD analysis of the sample heated up 480 °C revealed the formation of a single hcp-Mg phase, indicating that the second endothermic event was related to the decomposition of β-MgH_2_ phase.

These two endothermic reaction peaks were significantly shifted to the low temperature side to appear at 323 °C and 348 °C upon increasing the RBM time to 12.5 h, as elucidated in Fig. 3(b). Such significant decreasing of the decomposition temperature was related to an increasing of Ni volume fraction introduced to the milled powders (Fig. 3(d), leading to destabilize the MgH_2_ phases. After 25 h of RBM time, the two endothermic decomposition peaks were overlapped to disclose a wider endothermic peak appeared at a relatively low decomposition temperature of 218 °C, as displayed in Fig. 3(c).

#### Activation energy of dehydrogenation

In order to realize the effect of Ni catalysts introduced to the powders upon increasing the RBM time on the activation energy (E_a_) of MgH_2_ powders, individual DSC experiments were conducted with different heating rates (5, 10, 20, 30 and 40 °C/min) for the samples obtained after 3, 6, 12.5, 25, 100 and 200 h of RBM time. E_a_ of dehydrogenation related to each sample was calculated according to the Arrhenius equation:


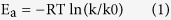


where k is a temperature-dependent reaction rate constant, R is the gas constant, and T is the absolute temperature. The E_a_ values were determined by measuring the decomposition peak temperature (T_p_) corresponded to the different heating rates (k) and then plotting ln(k) versus 1/T_p_. The E_a_ values were then obtained from the slope of line (-E/R, where R is the gas constant).

Figure 3(d) presents interesting relationships, showing the dependence of E_a_ on RBM time (red-line) and Ni concentration (blue-line) analyzed by two different techniques; namely EDS and inductively coupled plasma mass spectrometry (ICP-MS). Increasing the RBM time led to increase the number of ball-powder-ball collision, resulting an increase in the Ni particles worn away from the surface of Ni balls. Accordingly, MgH_2_ powders were continuously *in-situ* catalyzed by Ni that was monotonically increased during the first stage of RBM time from 0.8 wt.% (3 h) to 5.5 wt.% (25 h), as elucidated in Fig. 3(d). The Ni balls at the beginning of the RBM process had Mg powder-free-coated surfaces, as shown in the Supplementary Material (Fig. S2(a)). After 25 h–50 h of RBM time, the Ni-balls were coated by the soft Mg powders acted as Ni-wear resistant coats (Fig. S2(b)). This led to terminate the steep inclination of Ni mole fractions introduced to the milled powders to be about 7.2 wt.% (Fig. 3(d)). The MgH_2_ powders obtained during the second stage of RBM (25–50 h) tended to coat the Mg-layers adhered onto the surface of Ni-balls, as elucidated in Fig. S2(c). During the last stage of RBM, Ni concentration was almost saturated at values, ranged between 7.3 to 7.8 wt.%, as shown in Fig. 3(d).

The E_a_ of dehydrogenation was very sensitive to the changing of RBM time and Ni concentration, as indicated in Fig. 3(d). In fact, increasing the RBM time did not only lead to increase the Ni mole fraction introduced to the powders, but it also led to introduce sever lattice imperfections to the milled powders, leading to destabilize the MgH_2_ phase. These imperfections lead to disintegrate the Mg/MgH_2_ particles to form smaller crystallites that can facilitate better hydrogen diffusion with faster hydrogenation/dehydrogenation kinetics. The MgH_2_ powders obtained after 3 h–6 h of RBM time had large E_a_ values (140–120 kJ/mol), as shown in Fig. 3(d). After 12.5 h, E_a_ was sharply decreased to about 90 kJ/mole and reached to less than 75 kJ/mol and 69 kJ/mol for the powders obtained after 25 h and 50 h of RBM time, respectively (Fig. 3(d)). When the Ni concentration was terminated during the last stage of RBM (100 h–200 h), no significant changes on E_a_ could be seen, as displayed in Fig. 3(d).

For the purpose of the present study, a different milling runs, using FeCr-balls milling media were achieved under the same experimental condition to realize the effect of milling tool’s materials on E_a_. The concentrations of FeCr (presented as Fe) introduced to the powders upon collisions during the early stage (3–12.5 h) and intermediate (25–50 h) stages of milling were about 0.4 wt.% and 1.15 wt. %, respectively as shown in Fig. 3(e). We can realize that the improving of E_a_ seen during these stages of milling were mainly attributed to the graining refining process. Refining of MgH_2_ powders here led to slight decreasing in E_a_ from about 140 kJ/mol (3 h) to about 116 kJ/mol (50 h), as shown in Fig. 3(e). Further improving in E_a_ was attained upon increasing the RBM time to 100 h (~105 kJ/mol) and 200 h (~95 kJ/mol), as elucidated in Fig. 3(e). This obvious decreasing in E_a_ can be attributed to increasing of the FeCr concentrations that tended to reach relatively high values of about 1.6 wt.% and 2.26 wt.% after 100 h and 200 h of RBM time, respectively. Thus, we can conclude that when MgH_2_ powders were ball milled with the rather “soft” metallic Ni balls, a high abrasion taken place during the RBM process led to introduced a high concentration of Ni metal particles that played an important catalytic role for enhancing the decomposition of MgH_2_ at normal pressure.

### Enthalpy of hydrogenation and dehydrogenation

**Figure 4:**
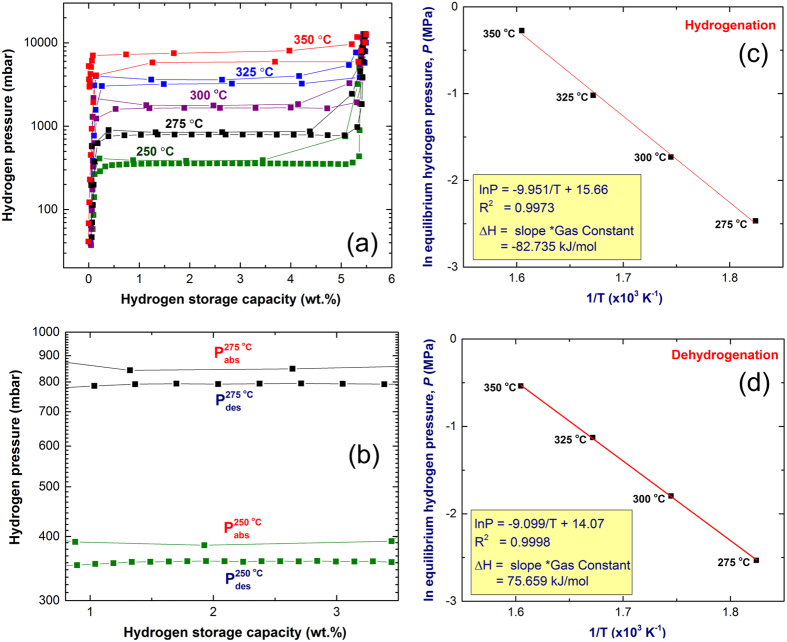
Pressure-composition-temperature (PCT) curves and van’t Hoff plot. The PCT curves of MgH_2_ powders obtained after 25 h of RBM time, using Ni-balls milling media are presented in (**a**) with different applied temperatures (250–350 °C). The PCT curves of the flat plateau extended from 0.8 to 3.5 wt.% H_2_ for the curves resulted with temperatures of 250 °C and 275 °C are displayed together in (**b**) in a different scale. The van’t Hoff plot of the plateaus presented in (**a**) for hydrogenation and dehydrogenation are displayed in (**c**) and (**d**), respectively.

The pressure-composition temperature (PCT) relations of MgH_2_ powders obtained after 25 h of RBM time, using Ni-balls milling media were volumetrically investigated by Sievert’s approach at different temperatures of 250, 275, 300, 325 and 350 °C, as elucidated in Fig. 4(a). The powders, which had a maximum hydrogen storage capacity of about 5.5 wt.% (Fig. 4(a)), possessed excellent PCT hydrogenation/dehydrogenation characteristics. This is implied by the near pressure values required for absorption, (P^abs^) and desorption (P^des^) at rather low temperatures of 250 °C (400/320 mbar) and 275 °C (870/780 mbar), as displayed in Fig. 4(b). Moreover, the powders manifested outstanding single-step hydrogenation/dehydrogenation behavior with negligible slope, as displayed in Fig. 3(e). Morover, a single reversible hydrogenation/dehydrogenation cycle was developed for each applied temperature. The presence of clear dehydrogenation plateaus can be seen in all temperature range. However, hydrogenation plateaus can be only seen in the range between 0.25–3.5, 0.25–4.3 and 0.25–4 wt.% H_2_ at 250 °C 275 °C and 300 °C, respectively as displayed in Fig. 4(a). Flat hydrogenation plateaus in the range of 0.15–4.35 and 0.05–5.4 wt.% H_2_ were realized at temperatures 325 °C and 350 °C.

The hydrogen equilibrium pressure measurements were used in the present study to investigate the heat of hydrogen absorption, using van’t Hoff equation:


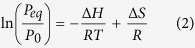


where P_eq_ is the hydrogen pressure under equilibrium at a given specific temperature, T, P_0_ is a reference pressure of 1 bar, R is the gas constant (0.0083145 J/K.mol), ΔH is the molar enthalpy of metal hydride formation (MgH_2_), and ΔS is the entropy of absorption. Thus, ΔH can be directly calculated from plotting the natural log of each P_eq_ point versus the corresponding 1/T, as shown in Fig. 4(b). In the present work, the calculated ΔH of hydrogenation for MgH_2_ obtained after 25 h of RBM time, using Ni-balls milling media was −82.72 kJ/mol.

The strength of Mg-H bonds, which can be expressed by the enthalpy of decomposition, can be also calculated by van’t Hoff approach, using the equilibrium dehydrogenation pressure in the PCT measurements. A van’t Hoff plot illustrating the relationship between ln(P) and 1/T for the decomposition of MgH_2_ powders obtained after 25 h is shown in Fig. 4(c). The ΔH was directly calculated from the slope of the curve presented in Fig. 4(d) and found to be 75.66 kJ/mol. Comparing this value with those ones of pure MgH_2_ reported by Reilly and Wiswakk (77.4 kJ/mol)^25^, and Klose and Stuke (81.86 kJ/mol)^26^ we can conclude that short-term of ball milling (25 h) MgH_2_ powders, using Ni-balls milling media, destabilized the chemically stable phase of MgH_2_, suggested by the rather decrease in the ΔH of decomposition.

### Hydrogenation/dehydrogenation kinetics behavior

#### Kinetics of hydrogenation

**Figure 5:**
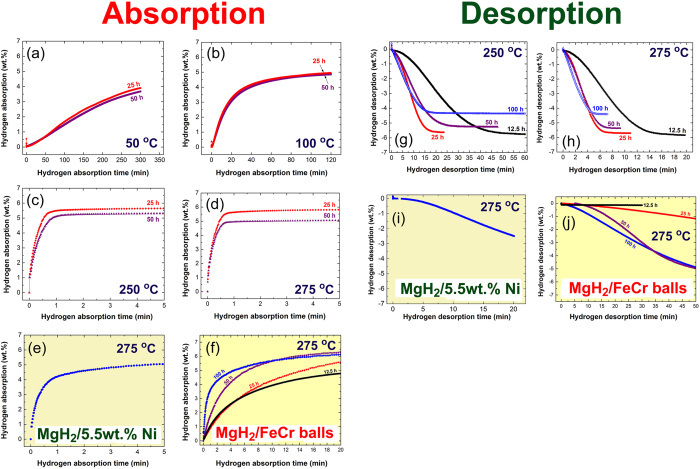
Kinetics of hydrogenation/dehydrogenation measured at different temperatures of MgH_2_ powders obtained after selected RBM time. Hydrogenation kinetics examined at (**a**) 50 °C, (**b**) 100 °C, (**c**) 250 °C, and (**d**) 275 °C for MgH_2_ powders obtained after 25 h and 50 h of the RBM time, using Ni-balls milling media. The hydrogenation kinetics of a different sample obtained after 25 h of RBM a mixture of MgH_2_ doped with 5.5 wt.% Ni nanoparticles is presented in (**e**) for comparison. Kinetics of hydrogenation for those samples obtained after different RBM time (12.5 h, 25 h, 50 h and 100 h), using FeCr-balls milling media are shown in (**f**). Dehydrogenation kinetics achieved at 250 °C and 275 °C for MgH_2_ powders milled with Ni-balls milling media for different RBM time (12.5, 25 h, 50 and 100 h) are shown in (**g**) and (**h**), respectively. The kinetics of dehydrogenation investigated at 275 °C for MgH_2_ powders doped with 5.5 wt.% Ni nanoparticles and then RBM for 25 h is shown in (**i**), whereas kinetics of dehydrogenation of MgH_2_ powders milled with FeCr-balls milling media obtained for different RBM time (12.5 h, 25 h, 50 h and 100 h), are presented in (**i**) and (**j**), respectively.

**Figure 6:**
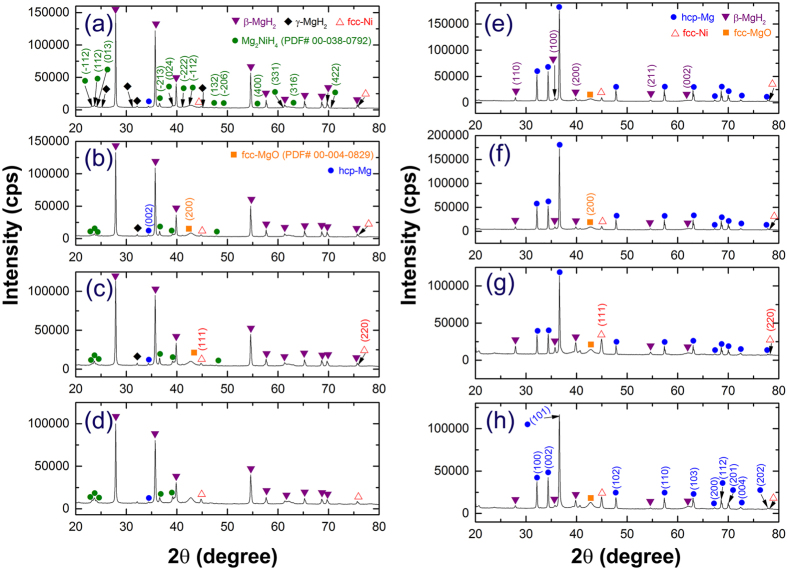
Structural changes of MgH_2_ powders obtained after 25 h of RBM upon subjecting to hydrogenation/dehydrogenation processes at different temperatures. XRD patterns of MgH_2_ powders obtained after 25 h and then subjected to hydrogenation process at (**a**) 50 °C, (**b**) 100 °C, (**c**) 250 °C, and (**d**) 275 °C. The XRD patterns of the samples obtained after 12.5 h, 25 h, 50 h and 100 h and then subjected to dehydrogenation process conducted at 275 °C, are shown in (**e**,**f**,**g** and **h**), respectively.

The hydrogenation kinetics behavior of MgH_2_ powders obtained after selected RBM time, using Ni-balls milling media were investigated at different temperatures in the range between 50 °C to 275 °C as displayed in Fig. 5(a) to (d). At very low temperature (50 °C), both 25 h and 50 h samples showed good hydrogenation capability for absorbing 3.9 wt.% and 3.6 wt.% H_2_, respectively within 300 min, as displayed in Fig. 5(a). The rather degradation on hydrogen storage capacity for 50 h sample is attributed to its higher Ni concentration (6.8 wt.%) when compared with the 25 h sample, which had 5.3 wt.% Ni (Fig. 3(d)). The XRD pattern of 25 h sample taken after hydrogenation process at 50 °C is shown in Fig. 6(a). The sample revealed a domain structure of β-MgH_2_ coexisted with small molecular fractions of γ-MgH_2_ and fcc-Ni. In contrast to the powders obtained upon RBM at ambient temperature (Fig. 1(b)), a minor molar fraction of Ni metal preferred to react with MgH_2_ powders during the hydrogenation process to form Mg_2_NiH_4_ phase (PDF# 00–038–0792), as shown in Fig. 6(a). Increasing the hydrogenation temperature to 100 °C led to a remarkable increasing in the hydrogen storage capacity for 25 h and 50 h samples to be 5 wt.% and 4.8 wt.%, respectively as displayed in Fig. 5(b). Such a moderate temperature increase led to a significant improvement in the hydrogenation kinetics, suggested by the short time required (120 min) for both samples to get their maximum storage capacity (~5 wt.% H_2_), as shown in Fig. 5(b). The X-ray analysis of the 25 h sample taken after the hydrogenation experiment at 100 °C indicated the formation of β-MgH_2_ coexisted with metastable β-MgH_2_ phases, as shown in Fig. 6(b). In addition to these phases, fcc-Ni and the reacted Mg_2_NiH_4_ phases were detected as well, as shown in the Fig. 6(b).

Figure 5(c) displays the hydrogenation kinetics behavior of 25 h and 50 h samples examined at 250 °C. After 0.5 min, 25 h sample absorbed about 5 wt.% H_2_, whereas the 50 h sample absorbed only 4.3 wt.% H_2_ (Fig. 5(c)). After 1.4 min, the 25 h and 50 h samples saturated at a hydrogen storage capacity of 5.5 wt.%, and 5.2 wt.% respectively, as elucidated in Fig. 5(c). These values did not show any changes upon increasing the absorption time up to 5 min. More improvement on the hydrogen absorption kinetics and storage capacity were attained for the 25 h sample when the hydrogenation temperature increased to 275 °C. The sample reached to its maximum hydrogen storage capacity (5.8 wt.%) after 2.5 min, as shown in Fig. 5(d). Same improvement can be realized for 50 h sample, however, its high Ni content lowered its hydrogen storage capacity to be about 5 wt.%, as elucidated in Fig. 5(d). Comparing these results with those obtained upon ball milling the MgH_2_ powders with 5.5 wt.% Ni nanoparticles for 25 h (Fig. 5(e)), we can conclude that our new approach of introducing of Ni particles gradually into the MgH_2_ matrix is considered to be the most prominent factor for the formation of homogeneous MgH_2_/Ni nanocomposite powders. Accordingly, this new catalyzation technique leads to superior hydrogenation kinetics even at low temperatures.

The XRD patterns of the sample milled for 25 h and then taken after hydrogenation process at 250 °C and 275 °C, are shown in Fig. 6(c) and (d), respectively. At such relatively high temperatures, the reacted Mg_2_NiH_4_ phase was grown, as suggested by the rather high intensity of their major Bragg peaks laid between scattering angle of 22° and 23° (Fig. 6(c) and (d)). We should emphases that the existence of this phase together with fcc-Ni may play a superior role for successful and fast dehydrogenation process, as will be discussed in the next section. In contrast to this sample, the hydrogenation kinetics of MgH_2_ powders obtained after for 25 h of RBM time, using FeCr-balls milling media revealed slower kinetics, suggested by the longer time required (5 min) to absorb 5 wt.% H_2_, as displayed in Fig. 5(e).

In order to understand the effect of Ni-particles introduced to MgH_2_ powders upon milling with Ni-balls milling media on improving the hydrogenation properties of MgH_2_, we have employed FeCr-balls milling media for preparing MgH_2_ powders under the same experimental conditions. Figure 5(f) shows the effect of RBM time on the hydrogenation kinetics of MgH_2_ powders achieved at 275 °C. However, the hydrogenation kinetics was improved with increasing the RBM time and FeCr content, the samples obtained after 25 h to 100 h required longer time (~20 min) to reach to ~6 wt.%, as shown in Fig. 5(i).

#### Kinetics of dehydrogenation

The dehydrogenation kinetics examined at 250 °C and 275 °C of the samples obtained after different stages of the RBM time (12.5, 25, 50 and 100 h) using Ni-balls milling media are shown in Fig. 5(g) and (h), respectively. The 12.5 h sample showed a poor kinetics at 250 °C, indicated by the very long time required (60 min) to reach its saturated hydrogen storage capacity (5.8 wt.%), as shown in Fig. 5(g). Significant improvement on its hydrogenation kinetics can be realized with increasing the hydrogenation temperature to 275 °C (Fig. 5(h)) when the 12.5 h sample released its maximum hydrogen storage capacity (5.8 wt.%) within 20 min only. The XRD pattern of this sample taken after the dehydrogenation process achieved at 275 °C (Fig. 6(e)) indicated that the powders consisted of hcp-Mg coexisted with Ni metal. Minor molecular fractions of β-MgH_2_ phase was also detected, as shown in Fig. 6(e).

The sample obtained after 25 h of RBM using Ni balls milling media showed a different dehydrogenation behavior when compared with the 12.5 h sample. It had the capability to desorb 5.8 wt.% within 21 min and 9 min at 250 °C and 275 °C, as elucidated in Fig. 5(g) and (h), respectively This fast desorption behavior can be attributed to its higher Ni content (5.5 wt.%) when compared with the 12.5 h sample (3 wt.%). Moreover, the presence of Mg_2_NiH_4_ phase resulted in the 25 h sample (see Fig. 6(c) and (d)) may also play an effective catalytic role for improving the dehydrogenation process. This phase could not be detected in the XRD pattern of the 12.5 h sample after hydrogenation process. The XRD pattern of 25 h sample after achieving a complete desorption at 275 °C is shown in Fig. 6(f). Beside those Bragg diffractions related to hcp-Mg, fcc-Ni and un-desorbed β-MgH_2_ is appeared. The absence of the reacted Mg_2_NiH_4_ phase formed during the hydrogenation process can be realized. This suggests a full decomposition of this phase into hcp-Mg_2_Ni and H_2_ during the dehydrogenation process. Moreover, the dehydrogenation kinetics behavior investigated at 250 °C (Fig. 5(g)) and 275 °C (Fig. 5(h)) of the samples obtained after 50 h and 100 h of RBM time using Ni-balls milling media did not show better kinetics when compared with 25 h sample. One drawback of further milling is the remarkable increase of unnecessary Ni particles introduced to the powders processed for 50 h (6.8 wt.%) and 100 h (7.6 wt.%), as can be seen in Fig. 3(d). Introducing of massive volume fractions of Ni to the MgH_2_ powders led to decrease the hydrogen storage capacity of 50 h and 100 h samples to about −5.3 wt.% and −4.4 wt.%, respectively as shown in Fig. 5(g) and (h). The XRD patterns of 50 h and 100 h sample examined after achieving dehydrogenation experiments at 275 °C are presented in Fig. 6(g) and (h), respectively. Both samples consisted of pronounced Bragg diffractions corresponding to hcp-Mg metals. This domain hcp-structure was coexisted with fcc-Ni metals that introduced to the powders upon using Ni-balls and small molar fractions of Mg_2_Ni alloy.

In contrast, the dehydrogenation process conducted at 275 °C for MgH_2_ powders milled with 5.5 wt.% of Ni nanoparticles for 25 h revealed poor kinetics, implied by the very long time (20 min) required to desorb about 2.5 wt.% H_2_, as shown in Fig. 5(i). The kinetics of desorption measure at 275 °C for those samples obtained after different RBM time, using FeCr-balls revealed poor dehydrogenation kinetics (Fig. 5(j)), indexed by the very long time (50 min) required for releasing −1 wt.% (25 h), and ~−5 wt.% (50 and 100 h) of hydrogen gas, as elucidated in Fig. 5(j).

#### Cycle-life-time

**Figure 7:**
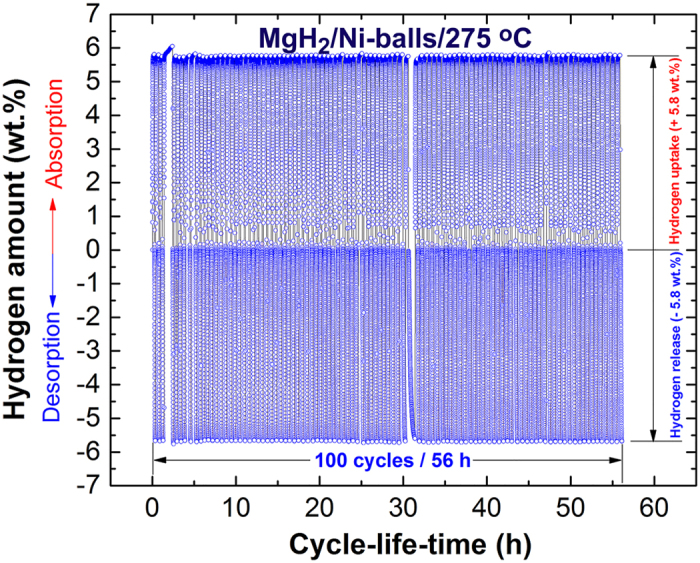
Cycle-life-time of MgH_2_ powders obtained after 25 h of RBM time. The cycle-life-time conducted at 275 °C for the sample obtained after 25 h of RBM time, using Ni-balls milling media.

There is no more fundamental than cycle-life-time examinations to characterize the capability of metal hydrides to achieve continuous cyclic hydrogenation/dehydrogenation processes. Successful metal hydride powders should maintain their hydrogen storage capacity without failure. In addition, the powders should show sustainable hydrogenation/dehydrogenation kinetics without serious degradation. In the present study, the MgH_2_ powders obtained after 25 h of RBM, using Ni-balls milling media was subjected to 100 hydrogenation/dehydrogenation cycles conducted for 56 h at 275 °C under a hydrogen gas pressure ranging between 10 bar (hydrogenation) 100 mbar (dehydrogenation). The powders were firstly activated by applying cyclic hydrogen gas sorption/desorption under pressure of 35 bar at 350 °C for 10 continuous cycles. This treatment is necessary for surface cleaning of the powders and to break down the oxide phase (MgO) formed on the powder surfaces. Figure 7 shows the hydrogen absorbed/desorbed cycles achieved continuously for 100 cycles at a temperature of 275 °C. No remarkable degradation in the hydrogen storage capacity could be detected even after 100 cycles (56 h), maintained at the level of 5.8 wt.%, as shown in Fig. 7. Moreover, the kinetics of hydrogenation/dehydrogenation processes remaining constant with no failure or decay.

## Discussion

Amongst the long list of spacious used as catalysts or additives for enhancing the poor hydrogenation/dehydrogenation behaviors of MgH_2_ powders, Ni is laid on the top of this list. This is attributed to its high performance for hydrogen splitting and its very low coast when compared with the noble metals such as Pd and Pt. It is therefore natural to expect not only the development of new Ni-based catalysts (e.g. Ni-Zr^15^, ZrPdNi^5^) but also the replacement of known noble-metal-based catalysts by reliable Ni and Ni-based analogs.

**Figure 8:**
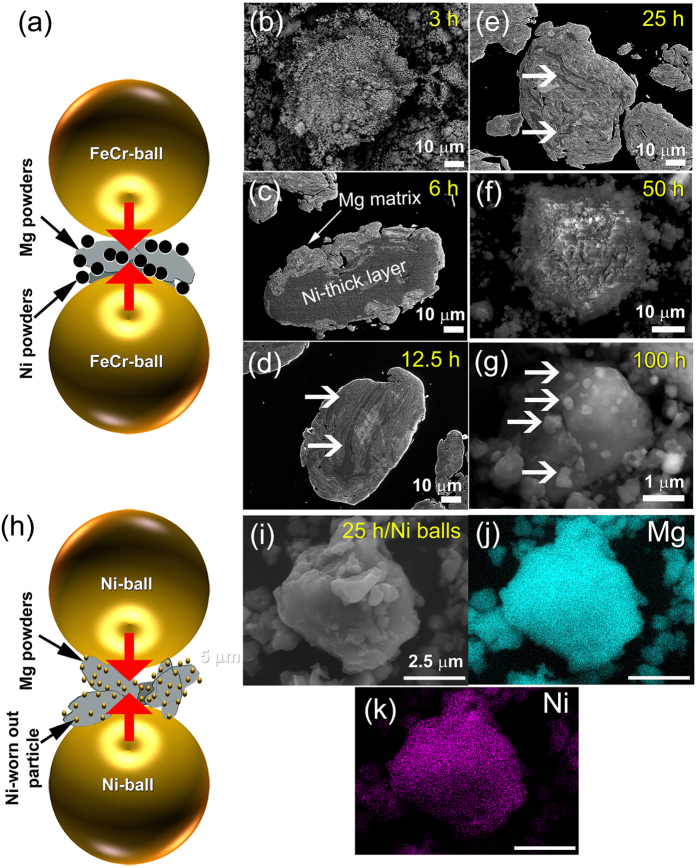
Mechanism of MgH_2_ catalyzation via RBM technique. (**a**) Schematic illustration showing the ball-powder-ball collisions during ball milling of Mg powders with 5.5 wt.% Ni particles, using FeCr. FE-SEM micrographs show the morphological characterizations of the composite powders obtained after **(b**) 3 h, (**c**) 6 h, (**d**) 12.5 h, (**e**) 25 h, (**f**) 50 h and (**g**) 100 h. The *in-situ* catalyzation mechanism, using Ni-balls milling media is schematically illustrated in (**h**). The FE-SEM micrograph of MgH_2_ powders obtained after 50 h is displayed in (**i**), whereas (**j**), and (**k**) refer to the corresponding EDS-elemental mapping of Mg and Ni, respectively.

Likewise many types of catalyst systems, catalyzing of MgH_2_ powders by Ni is usually carried out by “manual” doping the powders with the desired weight percentage of Ni particles/nanoparticles and then mechanically ball-milled (Fig. 8(a)) for certain time that can be extended to several hundred hours^13^,^15^. A major drawback of this traditional approach is the long milling time required to ensure a uniform dispersion of Ni into the MgH_2_ matrix. More serious disadvantage of this common process is the tendency of metallic Ni powders to form aggregates during the first few hours (3 h) of milling (Fig. 8(b)). Increasing the milling time (6 h) led the Ni powders to form thick-layered like metallography into the Mg-powders, as shown in Fig. 8(c). These layers were refined with increasing the ball milling time (12.5 h (Fig. 8(d)) resulting the formation of MgH_2_/thin Ni-layered composites, as shown in Fig. 8(e). At this intermediate of milling, the powders showed dramatic variation in their Ni contents from particle to particle and even within the particle itself. Toward the end of the milling process (50 h–100 h), the Ni layers were disintegrated into small particles adhered on the surface of MgH_2_ powders, as displayed in Fig. 8(f) and (g).

In the present study, and in contrast with the common mechanically-induced catalyization technique, we have proposed a new approach of gradual “*in-situ* catalyzation” of MgH_2_ powders during the ball milling process. Gradual introducing the Ni powders into the Mg/MgH_2_ powders found to be useful approach to overcome the formation of thick-Ni layers and to ensure the homogeneous distribution of Ni particles into the powder matrix. This alternative catalyzation method was carried out by high-energy ball milling of hcp-Mg powders under high pressure (50 bar) of hydrogen gas atmosphere, using Ni-balls milling media, as schematically illustrated in Fig. 8(h). The experiments were repeated for three independent runs to ensure the reproductivity of the results. After 3 h of ball milling, the Mg powder were agglomerated according to the cold welding effect during the milling process to form aggregates extended in sizes to about 60 μm in diameter (Supplementary Materials-Fig. S3(a)). Our results showed that at this early stage of RBM, the Mg powders (Fig. S3(b)) were homogenously catalyzed with Ni fine particles (Fig. S3(c)) that were worn out from the milling media (Ni-balls). Neither Ni-aggregates nor layers were formed at this stage of milling. However, the Ni powders dispersed into Mg matrix after this stage of milling was less than 1 wt.%, they were excellently distributed in the whole range of the matrix, as elucidated in Fig. S3(c). This is attributed to the absent of agglomerated Ni-layers that usually comes upon using a high content of metallic Ni powders. During the next stages of milling process Ni particles were gradually worn out from the Ni palls and hence dispersed into the MgH_2_ matrix, as suggested by the gentle increasing of Ni content with increasing the milling time shown in Fig. 3(d). Such gradual fashion of introducing Ni catalytic agent to the MgH_2_ powders led to form homogeneous composite powders with outstanding Ni particles distribution. During the first few hours of RBM Mg metal preferred to react with hydrogen gas to form β- and γ-MgH_2_ phases instead of reaction with Ni (low concentration), as shown in Fig. 1(c)). However, Mg_2_NiH_4_ phase is somehow showed better kinetics behaviors compared with MgH_2_, but its revealed unattractive hydrogen storage capacity (~3.5 wt.%^26^). The fine Ni particles introduced to the Mg metal matrix during milling process tackled under hydrogen gas atmosphere led to splitting the hydrogen molecules into hydrogen atoms, facilitating fast gas-solid reaction that completely achieved within 25 h (Fig. 1(c)). These fine Ni particles adhered onto the surface of MgH_2_ where occupied enormous sites (Fig. 2(c)) facilitating fast hydrogenation kinetics even at low temperatures (Fig. 5(a) and (b)). The hydrogenation/dehydrogenation kinetics were monotonically enhanced upon increasing the RBM time from 12.5 h to 50 h that led to improve the Ni particles distribution onto the surfaces of MgH_2_ powder particles. Such fine Ni particles played a vital role as grain growth inhibitors to maintain a short diffusion distance of hydrogen atoms along the MgH_2_ nanograins. Accordingly, the decomposition of MgH_2_ led to release the hydrogen atoms and formation of hcp-Mg was greatly enhanced (Fig. 5(g) and (h)). It has been pointed out by Isobe *et al*.^27^ that using Cr steel milling media for ball milling graphite powders led to introduce large amount of Fe contamination content (~11 wt.%) after milling for 80 h. The as-worn out Fe particles reacted with the milled graphite sample to form Fe_3_C. Existence of this phase led to enhance the hydrogenation/dehydrogenation kinetics of graphite powders^28^,^29^.

In summary, we have proposed a new approach for catalyzing MgH_2_ with Ni nanograins by high-energy reactive ball milling of Mg metal under 50 bar of hydrogen gas atmosphere, using Ni-balls milling media. Our results have shown that using Ni-balls had glorious benefits for improving the hydrogenation/dehydrogenation processes that were taken place very fast when compared with the as-doped MgH_2_ by Ni nanoparticles and with MgH_2_ powders milled with FeCr-balls. Our claims are based on the results shown here, which suggest that introducing Ni-particles into the Mg/MgH_2_ powders in a “gradual-dosing” fashion during the RBM process led to improve the homogeneity of the composite powders and maintained the nanocrystalline characteristics of MgH_2_ powders.

## Methods

### Preparation of MgH_2_ powders

Elemental Mg metal powders (~80 μm, 99.8% provided by Alfa Aesar - USA), and hydrogen gas (99.999%) were used as starting materials. An amount of 5 g Mg was balanced inside a He gas atmosphere (99.99%) - glove box (UNILAB Pro Glove Box Workstation, mBRAUN, Germany) and sealed together with fifty balls (11 mm in diameter) made of pure Ni metal (99.9 wt.%) provided by Wako, Japan (item# 144-07255, Lot # DPR1504) into a hardened steel vial (150 ml in volume), using a gas-temperature-monitoring system (GST; supplied by evico magnetic, Germany). The ball-to-powder weight ratio was maintained at 40:1. However, in a parrel experiments Cr-steel balls (11 mm in diameter), using the same ball-to-powder weight ratio, were used as milling media under the same experimental conditions. The vial was then evacuated to the level of 10^−3^ bar before introducing H_2_ gas to fill the vial with a pressure of 50 bar. The reactive ball milling (RBM) process was carried out at room temperature, using a high-energy ball mill (Planetary Mono Mill PULVERISETTE 6, Fritsch, Germany). The RBM process was interrupted after selected milling time (3, 6, 12.5, 25, 50, 100 and 200 h) where the vial was opened inside the glove box to take a small amount (~300 mg) of the milled powders for different analysis. Then, the RBM process was resumed, using the same operational conditions shown above.

### Powder characterizations

XRD AND HRTEM. The crystal structure of all samples was investigated by XRD with CuKα radiation, using 9 kW Intelligent X-ray diffraction system, provided by SmartLab-Rigaku, Japan. The local structure of the synthesized material powders was studied by 200 kV-field emission high resolution transmission electron microscopy/scanning transmission electron microscopy (HRTEM/STEM, supplied by JEOL-2100F, Japan), which is equipped with Energy-dispersive X-ray spectroscopy (EDS) supplied by Oxford Instruments, UK. In addition to the elemental analysis achieved by EDS approach, we employed ICP technique to get the elemental analysis by a chemical analytical approach.

#### Thermal stability

Shimadzu Thermal Analysis System /TA-60WS-Japan, using differential scanning calorimeter (DSC) was employed to investigate the decomposition temperatures of MgH_2_ powders with a heating rate of 20 °C/min. The activation energy for of the powders obtained after different RBM time were investigated, using Arrhenius approach with different heating rates (5, 10, 20, 30, 40 °C/min).

#### The hydrogenation/dehydrogenation behaviors

The hydrogen absorption/desorption kinetics were investigated via Sievert’s method, using PCTPro-2000, provided by Setaram Instrumentation, France, under hydrogen gas pressure in the range between 200 mbar (for dehydrogenation) to 10 bar (for hydrogenation). The samples were examined at different temperatures of 50, 100, 250, and 275 °C. In the PCT measurements, the dosed pressure in absorption/desorption was gradually increased/decreased by 1000 mbar until equilibrium pressure reached to 13000 and 50 mbar, respectively. The PCT absorption/desorption kinetics were fitted in real-time by the HTPSwin software, to determine the sufficient equilibration time (the next point would start when the uptake had relaxed just 99% to asymptote). A minimum time of 30 minutes per equilibrium point and a maximum timeout of 300 minutes were set for each kinetic step in both the absorption and desorption isotherms.

## Additional Information

**How to cite this article**: El-Eskandarany, M. S. *et al. In-situ* catalyzation approach for enhancing the hydrogenation/dehydrogenation kinetics of MgH_2_ powders with Ni particles. *Sci. Rep.* **6**, 37335; doi: 10.1038/srep37335 (2016).

**Publisher’s note**: Springer Nature remains neutral with regard to jurisdictional claims in published maps and institutional affiliations.

## Supplementary Material

Supplementary Information
