# Supplementary material for: In-situ catalyzation approach for enhancing the hydrogenation/dehydrogenation kinetics of MgH2 powders with Ni particles
Source: Sci Rep. 2016 Nov 16;6:37335. doi: 10.1038/srep37335 (PMC5110972; doi:10.1038/srep37335)
Supplement: Supplementary Information [file srep37335-s1.doc]

**Supplementary Information**

**In-situ catalyzation approach for enhancing the hydrogenation / dehydrogenation kinetics of MgH_2_ powders with Ni particles**

M. Sherif El-Eskandarany*, Ehab Shaban, Naser Ali, Fahad Aldakheel, Abdullah Alkandary

Nanotechnology and Advanced Materials Program,

Energy and Building Research Center, Kuwait Institute for Scientific Research

Safat 13109, Kuwait - State of Kuwait

*Correspondence author

Tel.*: ‏+(965) 24989265;* Fax: *+(‏965) 24956609*

E-mail address: primary: msherif@kisr.edu.kw

Secondary: msherif99@yahoo.com


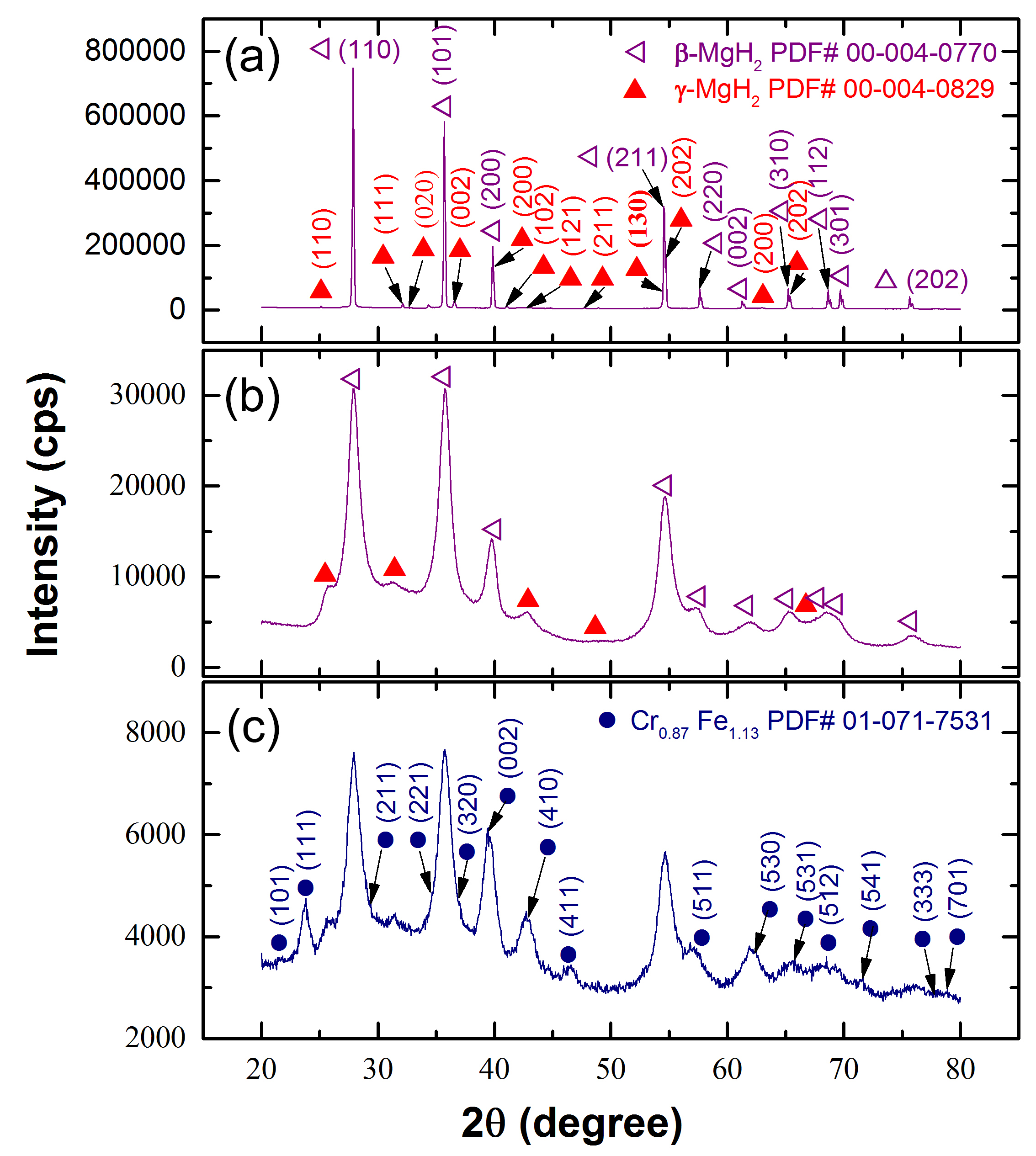


**Supplementary Figure-S1**

XRD patterns of (a) pure hcp-Mg powders ball-milled under hydrogen gas pressure (50 bar) for (b) 25 h, and (c) 50 h of RBM time.

**
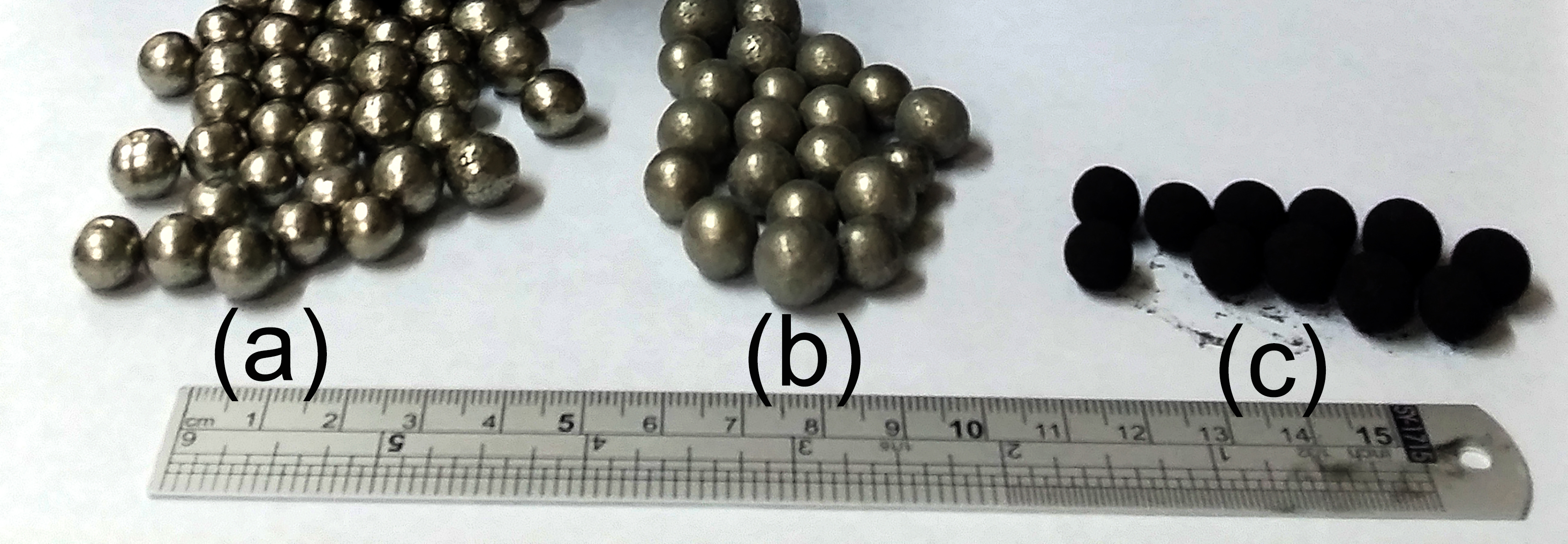
**

**Supplementary Figure-S2**

Photo taken for the Ni balls used as milling media after reactive ball milling for (a) 0 h, (b) 6 h, and (c) 25 h of milling


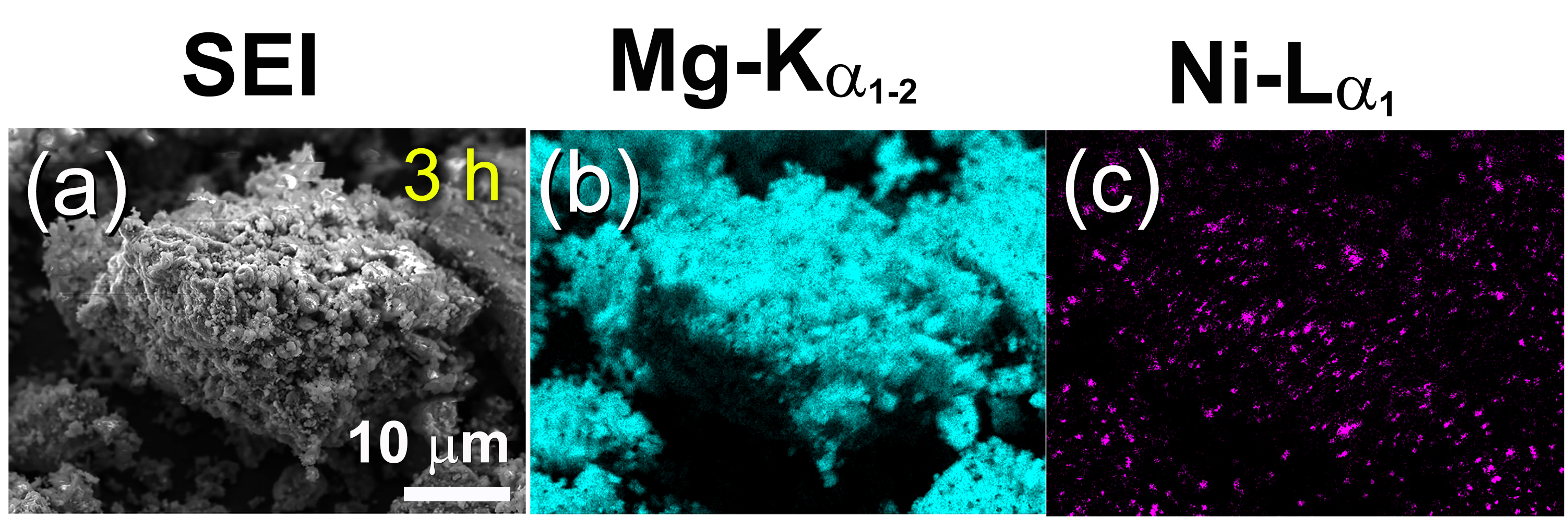


**Supplementary Materials-Figure S3**

(a)SE image, and EDS-elemental mapping of (b) MgK_a1-2_ , and (c) Ni-L_a1_ of MgH_2_ powders obtained after 3 h of RBM time, using Ni-balls milling media.
